# Recollections of a Journey Through a Psychotic Episode: Or, Mental Illness and Creativity

**DOI:** 10.4103/0973-1229.32163

**Published:** 2007

**Authors:** Anonymous

## Sat Jul 30, 2005 9:18 pm

My high school experiences were marked by excellence in academics and in sports. I was Valedictorian of my class and I amassed a record of 42 wins and seven losses in dual meet competition in wrestling. I was very competitive both in academics and in sports. My one romance was with the girl next door. We are still friends after 40 years but she married a great guy and I am friends with both of them. The one-busted affair I had resulted in severe depression and may have influenced my mood swings years later.

There were no precursors of my bipolar personality, although my aunt is bipolar and my father is unipolar depressed. It was triggered by major insights in plate tectonics. Shortly after I made these discoveries, my academic friends noticed a change in my personality. They said that I had become a “biggee” i.e., they recognized that I was rapidly stepping into grandiosity. As the mania stepped in, I couldn’t eat, I couldn’t sleep, I threw up and the racing of thoughts was mirrored by a racing of my libido, something Kay Redfield Jamieson alludes to. Finally, I drove out to my parent’s house and there my father fed into the mania in a way my mother thought was wonderful i.e., we appeared to be having deep, meaningful discussions about physics. One of the insights I had was the concept of extropy, which I define as, “Whenever a system has more energy than it can expel along a path of least resistance, it must expel the energy along a path of greater resistance.” When I discussed this concept with a chemistry professor, his response was, “Whenever a system has more energy than it can expel along a path of least resistance, it must expel the energy along a path of less than least resistance.” Needless to say, I was confused. How can there be a path of less than least resistance?

The next morning when I tried to influence the 1980 Presidential election by getting in touch with the Science Advisor to the President, my brother alluded as how I needed help. By now, my parents had seen enough and decided to drive me into the Capital District Psychiatric Centre. On the way there I had the horrible perception that I had taken the next step in the evolution of humanity and that the first action of this new subspecies of humanity was to wipe out the existing human race, as had been the case with previous hominids. When this realization hit me, I became hysterical and started crying uncontrollably and ordered my mother to stop the car. Then in a very soothing tone of voice she said, “If you really are the next step in the evolution of mankind, you will have the wisdom to assimilate rather than destroy the human race.” This calmed me down. The rest of the trip was uneventful.

That is the first phase of the experience.

## Sun Jul 31, 2005 9:15 pm

This is the second part of my narrative. The first dealt with events leading up to my stay at the Capital District Psychiatric Centre.

When I got out of the car at CDPC I was highly agitated. I had ordered my parents to take me into the FBI for questioning. (In my mind, I was a threat to society). At CDPC I was admitted under emergency guidelines i.e., two admitting psychiatrists had to evaluate me. (This was unnecessary; I would have been willing to sign myself in voluntarily). The doctor reminded me of Wilfred Brimley who does Quaker Oats commercials in the states. I ordered him to take me to the FBI for questioning. When he was done with the interview, I remember being mildly amused that he was getting his blood pressure taken. My attitude was- I’m the one who needs help; why is he getting his blood pressure taken? The second psychiatrist was a woman who didn’t like taking orders but she agreed that I should be admitted on an emergency basis.

Next, I was given a magical pill call Thorazine. It totally knocked me on my ass. I mean I was unconscious and woke up in a small dark room off to the side of the hallway. My mother was hovering nearby as I slowly regained consciousness. When I was fully recovered, I was so angry no one had called the FBI as promised that I made a mad dash for freedom. I was tackled by the attendants but not before taking a swing at a cop.

Then I was taken to Unit J.

I knew that I was in a psychiatric ward, but didn’t know why. I couldn’t understand why no one could think as fast as I was thinking. Then I began to realize why I had such strong feelings about the presidential elections. It was a classic battle of good versus evil. This was 1980 and the election was Jimmy Carter versus Ronald Reagan. Jimmy Carter’s initials are JC as in Jesus Christ. What do we have to connect Ronald Reagan with evil? Regan was the name of the little girl in the Exorcist who was possessed by the devil. So, in my mind, we had a classic battle of good versus evil. I was constantly looking for signs at this time and when I picked up a scrap of paper on the floor for no particular reason, it had only one word on it-Plains, as in Plains Georgia where Jimmy Carter was born.

I was being gregarious, shaking hands with people, patting them on the back etc. Then, I approached this woman gently rocking herself next to a wall. I went to touch her shoulder and she said, “Don’t touch me.” But it was too late and I touched her. At that instant I felt an overpowering shock that almost knocked me off my feet. My immediate reaction was, “Oh my God. I just touched Satan”. What was it? Perhaps just a powerful static discharge in a hypomanic state. But I fought to control my emotions and immediately went up to the front desk and said, “Boy, I just touched that woman over there and she got upset”. You will note that I made no reference to Satan because I knew this would be a bad idea. Her response, “We have very sick patients here, don’t do that again”.

Now, I knew why I was at CDPC; I was there to do battle with Satan. So all I did from that point onward was wander through the centre wondering in what shape the battle would take place. After about 15 minutes to half an hour, I noticed this small woman acting peculiarly. My immediate reaction was, “Satan’s got her.” So I froze, not moving a muscle. I wanted to see what would happen next. Remember, I am a scientist and I thought I was in the middle of paranormal phenomenon and wanted to do a good job observing. The woman headed to the lower lounge in what I would call a sleep jog. Then she abruptly turned right and headed towards me. I tensed up. She proceeded to circle me in a clockwise tightening spiral and then headed off. It made me very nervous when she was in back of me. I was afraid she might stick a knife in my back. My reaction was, “That’s it? Circle me a few times then head off?” I was disappointed. Then she headed back to where she started, paused for a few seconds and then did the same routine i.e. head for the lower lounge and then abruptly head towards me. This time she circled me two to three times, brushed my left shoulder and stood in front of me with her hands slowly reaching up. It was as though she had a pot filled with water that she was putting on a high shelf. Her hands rose to the level of my throat and, very slowly and carefully, she put her hands around my throat and began to squeeze. Naturally, if there was any doubt in my mind that I was doing battle with Satan, having one of the patients try to strangle me right on cue, made a believer out of me. The pressure she exerted on my throat was comparable to a woman’s firm handshake.

My reaction to being strangled was to sit there and try to figure out what to do next. Then it occurred to me that I could improve on the observation by having one of the staff members observe it, so I turned to the front desk very slowly and said, “Hey Fred look, Jane’s trying to strangle me”. (These are not their names). He responded, “No she’s not, she’s just trying to hug you”. What had happened is that when I turned to the front desk, my throat twisted in her hands, she let go of my throat. So as her hands were dropping to her side, the staff member only saw her hands dropping to her side and he simply said it looked like she was trying to hug me.

Next, I performed an “exorcism” on this woman, which completely devalued any other experience I had that night, simply because any schizophrenics might assume I was trying to kill their boss, so I would become a lightening rod for norm violating behaviour.

For whatever reason, I seem to have two buttons in my brain. Push one button, Satan pops out; press a second button, it says kill Satan. I attempted to kill Satan by standing in front of this vinyl curtain separating the TV room from the main lounge. Then I felt my arms pulled into the shape of the cross and as I was standing there, I began to chant over and over, “Come through the wall of time Satan. Die Satan. Die. Come though the wall of time, Satan. Die Satan. Die”. As I did this, I could feel a cool breeze coming through the plastic curtain and I just about passed out and collapsed into a chair. I felt that as I pulled Satan through the wall of time I was killing him.

What was really going on? Norm violating behaviour is routine in a psychiatric centre, so the woman strangulating me could just be coincidental. Or it might have been a delusion if the staff member was correct. When I stood in front of this woman just prior to doing an “exorcism”, I could feel the hairs on the back of my neck stand up. I was full of rage and hatred at this strange animal presence I felt within her. At first this felt good and then I remembered the movie *Star Wars* and how dangerous the dark side of the force was, so I quieted down before I did the “exorcism”. Basically, I just told Satan, “Leave that poor woman alone”. I said it a few times and the woman grabbed my hand and the “exorcism” was over. The episode in front of the plastic curtain-delusions supplemented with hyperventilation.

Part way through the evening one of the patients I had befriended came up to me in a very agitated manner and I immediately was defensive. Then he said, “I’m all right, Alex”. (*Name changed -eds.*) And I knew he was ok. Then he asked me a very bizarre question. He asked me to clean up his room, the site of a recent suicide and he asked me to clean up bloodstains and straighten out the clothes’ rod in this room. This seemed like a reasonable request so we went up to his room and there were blood stains i.e. dark brown spots on his rug. So I went into the shower room and got shampoo to try to clean up the rug (without much success-the stains had really set). Then I went into his closet and put my shoulder against the rod, which was square shaped and hollow and forced it back into place.

One of the things that bothered me was it looked like a murder scene, not a suicide. If we assume that the patient killed himself by committing suicide and hanging himself from the clothes’ rod, wouldn’t you expect the rod to be bent in a vertical plane? Instead, the rod was bent at a 45-degree angle. How is this consistent with suicide? When I asked my caseworker about this he let it slip, “If you had seen the position of the body”. I informed the Albany police that it looked like murder, but I was ignored.

When I was at CDPC, I was ravenous and ate double portions of food. I was not exercising and I was not gaining weight. I must assume that my brain was consuming as many calories as a full-grown man would consume for his entire body. This was even when I was typing 10–12 hours a day. The staff got me a typewriter and I typed a 73-page magnum opus on geology. When I circulated it at the State University at Albany, it bombed although one world-class geologist said that much of it had been said before, so it wasn’t as new as I thought it was. He suggested I submit it in pieces because he acknowledged that he had difficulty getting his major works published.

This concludes my second instalment.

## Mon Aug 1, 2005 7:43 am

This is the third part of my narrative.

Just before I was discharged from CDPC, I appeared in front of a panel of psychiatrists to determine if I was suitable to be discharged. There was a classic exchange between one of the psychiatrists and me. (Later I learned that there had been a real battle between caseworkers and this psychiatrist. They wanted me to be classified as bipolar and he wanted to classify me as schizophrenic. I am thankful that the caseworkers prevailed). In a thick accent he asked me, “Meester Alex. How do you define modesty?” Without hesitation I replied, “Modesty is the ability to do what one says one can do”. He then asked, “How do you define grandiosity?” I replied, “Grandiosity is the inability to do what one says one can do”. When Muhammad Ali boasted, “I am the greatest!” was he being modest or grandiose?

My trip home was uneventful and I settled into a routine, but then something terrible happened. I descended into a pit of unimaginable evil. Out of nowhere, I experienced a level of evil and perversity that defies the imagination. It was on a par with having the top of my head cut off with a hacksaw and having someone pour in a bucket of raw sewage. That’s not the evil part; the evil part is I enjoyed it. Which gets back to CDPC. Remember that time in front of the vinyl curtain when I summoned Satan through the wall of time into me. It was exactly like the scene in the *Exorcist* just after Father Merrin died and Father Karras said to Regan, “Take me!” And the devil left Regan and entered Father Karras and he leaped through a window to his death. I had summoned Satan through the wall of time and he entered me (or so it seemed at the time). But I suffered no ill effects at the time. It wasn’t until much later that I had these evil perceptions.

We have been led to believe in the age-old debate-heredity versus the environment. What if this is too simplistic? Suppose there is an even more important source of input-spirituality? Is our spiritual component just as important as our heredity and the environment? In my case it was uncontrollable spirituality that led me to melt down and need help. Later it was negative spirituality that caused me to descend into evil. One thing is clear: 1) Nothing in my genetic makeup correlates with the evil I experienced, 2) Nothing in the environment correlates with the evil. So where did it originate?

I can remember how this episode started, but I cannot remember how it ended. I do remember standing in front of a mirror in the bathroom and seeing this hideous face staring at me from the mirror. It so traumatized me that I shaved my beard the next day. Since then, I’ve re-grown my beard.

Nothing lie this phase of evil has ever happened to me before or since. I don’t know the cause; all I know is that it was horrible. Since then I have been institutionalised twice, but the stays were unremarkable compared to the first episode. Initially, I fought the medication after the first episode (I was hooked on the mania); but I think the evil episode may have shepherded me into treatment. I have been on lithium therapy for 25 years and have had episodes about 10 years apart. Prior to the second episode, I was screwing up my meds because no one gave me a pill dispenser so I could keep track of my meds. I was under-dosing and over-dosing myself periodically. Before my third episode, I had stopped taking one of my meds about three months before my episode.

This concludes the third part of my narrative.

## Mon Aug 1, 2005 4:54 pm

This is part four of my narrative. This is the last part dealing with my obvious delusional phases and will be superseded by the discoveries that were made after I returned to “normal”.

After my first episode, I fought the medication because I liked my creativity and I didn’t care that my behaviour took a real toll on my family. Twice I went swimming in 50-degree water fully clothed because my demons were out of control. Then I learned that cold water is a primitive form of shock therapy. When I got out, a warm shower allowed me to return to normalcy, at least for an hour or so and then the demons reappeared.

One of the characteristics of the delusional phases is that I looked for signs, omens and portents in everything. For example, when I had delusions of being very important, I ordered God to destroy the house above ours with a tornado. I shouted at the sky, “Get that house out of here. Get it out now!” Now for the amusing part. A few years later I moved into a house that had been hit by a tornado!

Once when I went to the dentist, I didn’t want the dentist to know I had a history of mental illness, so I lied on my medical history. I got nitrous oxide and bam! Suddenly I was in the middle of a battle with Satan. I wasn’t delusional before the nitrous oxide, but got real delusional after getting it. It went away as soon as I got off the nitrous oxide. Next time I went to the dentist and again got nitrous oxide and a second time I became delusional. On the third time I asked myself which is it going to be? Do battle with Satan or get a shot of novacaine? I decided to do battle with Satan. As you can see, I don’t regard doing battle with Satan as a big deal. But on the third episode I got scared. The delusions lasted for several hours after I was off nitrous oxide. It is one thing to do battle with Satan over the length of time of the dentist visit; it is entirely different to be delusional for several hours afterwards. That was the last time I got nitrous oxide.

One of the experiences I have had does not bode well for the religious community. One of the insights I had was that things are not what they appear to be. My interaction with the paranormal realm at first led me to believe everything was as it appeared to be i.e. when I thought I was dealing with God, it was God and when I thought I was dealing with Satan, it was Satan. Then I had this insight, that all was not as it appeared to be, that God was testing my great intelligence by appearing to me as Satan. When I had this insight, my head filled with laughter and all these voices commended me on my brilliance for this deduction. I shook my fist at the sky and said I’ll get even with you guys when I get up there. I was very pleased with this deduction.

Something kept gnawing at me-there was something fundamentally wrong with God appearing as Satan. And then it hit me. God would never appear as Satan because that would destroy communication of God with humanity. So what did I experience? Very likely, Satan appeared to me as God. This is why the prophet system has no hope of success. If every two-bit prophet who came down the pike could see through Satan’s deceit, Satan would be a pussycat. My grandmother could beat up on him. But Satan is an opponent worthy of God; he eats up prophets like popcorn. Men who don’t have a clue run the entire prophet system whether they are dealing with God or Satan.

## Mon Aug 1, 2005 10:44 pm

I saw my psychiatrist last week and he is very pleased with my progress. I have experienced no racing of thoughts and no grandiosity recounting my tales. It’s been cathartic but is otherwise harmless. If you wish to collect and codify my experiences i.e. put your own spin on my experiences, feel free to do so. I didn’t tell my psychiatrist about much of this stuff because I like being diagnosed as bipolar. I was afraid if I told them about this stuff I might be reclassified as schizophrenic-and change my medication.

## Tue Jul 26, 2005 12:15 am

The ultimate act of insanity is sanity. Mental illness is not the source of enlightenment, what it is, is a way to get beyond the dimensions that confine us. We are boxed in by four dimensions; when the mentally “ill” individual is exposed to the right stimulus, their minds are free from the confines of these dimensions. Once they break those bonds and get to experience higher dimensions the need for delusional/psychotic episodes is ended and their need for additional episodes is lessened. Clearly, an individual who never recovers from psychosis is lost to the gene pool because they cannot communicate what they have learned and it is unlikely they will procreate (unless, of course, they have sex in a delusional state). Thus, just as it is absolutely necessary to experience these delusional episodes in order to access higher dimensions, it is critical that the delusional episode ends so that the individual can express his/her views in a calm and orderly manner. Don’t forget-the delusional individual is around family and friends typically and they will need a long time to trust the judgment of the individual. Thus, any wisdom or insights they have gained will be viewed suspiciously. It takes time and patience for the delusional individual to convey the fruits of their experiences.

Are delusional episodes necessary for evolution? Absolutely - at least for now. Maybe in the future, some people can access higher dimensions at will and, for them, mental alternatives will be obsolete. For now, they offer the best alternative for intellectual growth of our species. The real danger in all this is some genius i.e., biologist/psychiatrist may pinpoint a certain gene in our genome that correlates with manic depression and schizophrenia and these foetuses are selectively aborted. This would be a huge hit in our evolutionary progress.

## Tue Jul 26, 2005 6:59 pm

Rarely has anything I’ve experienced in a delusional state been worthwhile. The one exception: I am an average chess player, but on three occasions I have had “competitive intuition”. So, when I was delusional with competitive intuition I called a Grandmaster who gives me lessens and challenged him to a chess game over the phone. Normally, he would beat me 99 games in a row and I might get one draw in a hundred games. In this game I was winning but he offered me a draw in a poorer position and I accepted it (I didn’t want to get greedy). This is the second Grandmaster I’ve played; in a simultaneous game against a Grandmaster, I had the better position, but lost. So in two games I’ve drawn one game and lost the second.

**My creativity goes through stages:**

1. Insights marked by an increase in intuition;
2. Increased excitement;
3. Delusional episodes marked by grandiosity and rapidity of thought. This is what I call the fact assumption spiral i.e. assumptions become facts, facts are used to generate assumptions; these assumptions then become facts. One of the key indices for me when I experience an episode is that I stop challenging my assumptions;
4. Massive insights of dubious value;
5. Paranormal delusions;
6. Decline and consolidation of thinking;
7. Return to normalcy;
8. Bona fide insights of value.

The final stage lasts 50–100 times the length of the rest of the stages combined, leading to the following question, “If stages 1–7 are necessary to get to state 8 is it worth it?” Bear in mind that for every full-blown episode, there are mini episodes that don’t result in a full blown delusional state.

One question I have asked myself is, “What kind of person would I have become if I had lived 10,000 years ago? Would I have been an outcast left to fend for myself or would there have been caregivers 10,000 years ago?” Two things are clear to me:

1. My illness probably correlates with my creativity;
2. Without the discipline of psychiatry, my demons would probably have destroyed me.

